# SALL4 in gastrointestinal tract cancers: upstream and downstream regulatory mechanisms

**DOI:** 10.1186/s10020-024-00812-z

**Published:** 2024-04-08

**Authors:** Tairan Wang, Yan Jin, Mengyao Wang, Boya Chen, Jinyu Sun, Jiaying Zhang, Hui Yang, Xinyao Deng, Xingyue Cao, Lidong Wang, Yuanyuan Tang

**Affiliations:** 1 School of Basic Medical Sciences, Xinxiang Medical University, Xinxiang, 453003 China; 2 First Clinical Medical College, Xinxiang Medical University, Xinxiang, 453003 China; 3 State Key Laboratory of Esophageal Cancer Prevention & Treatment and Henan Key, Laboratory for Esophageal Cancer Research of The First Affiliated Hospital, Zhengzhou University, Zhengzhou, 450052 China

**Keywords:** SALL4, Gastrointestinal tract cancer, Upstream regulation, Downstream target

## Abstract

Effective therapeutic targets and early diagnosis are major challenges in the treatment of gastrointestinal tract (GIT) cancers. SALL4 is a well-known transcription factor that is involved in organogenesis during embryonic development. Previous studies have revealed that SALL4 regulates cell proliferation, survival, and migration and maintains stem cell function in mature cells. Additionally, SALL4 overexpression is associated with tumorigenesis. Despite its characterization as a biomarker in various cancers, the role of SALL4 in GIT cancers and the underlying mechanisms are unclear. We describe the functions of SALL4 in GIT cancers and discuss its upstream/downstream genes and pathways associated with each cancer. We also consider the possibility of targeting these genes or pathways as potential therapeutic options for GIT cancers.

## Introduction

Gastrointestinal tract (GIT) cancers, including esophageal cancer (EC), gastric cancer (GC), colorectal cancer (CRC), liver cancer, and pancreatic cancer, are major causes of morbidity and mortality globally (Shoji et al. 2022; Abdul-Latif et al. 2020). In 2020, CRC, GC, and EC ranked fifth, sixth, and seventh, respectively, among 36 cancers in terms of new deaths (Sung et al. 2021). Although multiple nonsurgical treatment strategies have been employed, they are limited because of side effects (Johdi and Sukor 2020) and cancer resistance (Huang and Yu 2018; Sahin et al. 2019). Therefore, developing new therapeutic targets is essential.

Spalt-like transcription factor 4 (SALL4), a cancer stem cell (CSC) marker (Islam et al. 2015), has been identified as a promising biomarker and diagnostic/therapeutic target owing to its overexpression in various cancers, including GIT cancers, with adverse progression and poor outcomes (Zhang et al. 2015; Sun et al. 2022; Dirican and Akkiprik 2016; Yong et al. 2013). SALL4 is excessively expressed in various malignant tumors, such as esophageal cancer (He et al. 2016), gastric cancer (Wang et al. 2021a; Yang et al. 2021a; Shao et al. 2020), colorectal cancer (Ardalan Khales et al. 2015), hepatocellular carcinoma (HCC) (Yong et al. 2013), breast cancer (Dirican and Akkiprik 2016; Yang et al. 2022), lung cancer (Kobayashi et al. 2011; Li et al. 2020) and acute myeloid leukemia (AML) (Wang et al. 2016). SALL4 plays a vital role in regulating the cell cycle and apoptosis, as well as in the formation and development of malignant tumors, but its role in different tumors is regulated by different mechanisms (Sun et al. 2022; Hwang et al. 2020; Liao et al. 2018). The function and mechanism of SALL4 in gastrointestinal tract cancers require further investigation, which may provide new insights into tumor therapy.

In this review, we summarize current research data on the roles of SALL4 in gastrointestinal tract cancers and the underlying mechanisms. We also describe the upstream regulatory genes (Table 1) and downstream target genes/pathways (Table 2) of SALL4 in each GIT cancer.

**Table 1:** Upstream regulatory genes of SALL4 in GIT cancers

Gene	Tumor	Function in the tumor	Action on SALL4	Mechanism	References
NRBP1	ESCC	Reduces tumorsphere formation	Degrades	Drives ubiquitination	Hwang et al. (2020); Liao et al. (2018); Wilson et al. (2012)
THG-1	ESCC	Promotes tumorsphere growth	Suppresses degradation	Antagonizes NRBP1	Hwang et al. (2020); Zargari et al. (2020)
MEIS1	ESCC	Related to self-renewal	Promotes expression	Unconfirmed	Zargari et al. (2020)
ILF2	ESCC	Enhances stemness and tumor-initiating capacity	Promotes stabilization and expression	Facilitates nuclear mRNA export, inhibits degradation	Li et al. (2021)
KDM6A	GC	Promotes progression	Promotes expression	H3K27me3 demethylation	Ren et al. (2023)
EZH2	GC	Inhibits progression	Suppresses expression	H3K27me3 methylation	Ren et al. (2023)
YAP	GC	Induces proliferation and self-renewal, protumor effect	Promotes expression	Unconfirmed	Bie et al. (2020)
miR-188-5p	GC	Promotes proliferation and migration	Promotes expression	Induces transcription of SALL4	Wang et al. (2019a)
miR-16	GC	Inhibits proliferation and migration	Suppresses expression	Directly targets SALL4	Jiang and Wang (2018)
miR-3622a-3p	CRC	Exerts antioncogenic effect	Suppresses expression	Directly targets SALL4	Chang et al. (2020)
miR-219-5p	CRC	Inhibits survival, migration, invasion and drug resistance	Suppresses expression	Directly targets SALL4	Cheng et al. (2015a)
miR-508	CRC	Blunts EMT, stemness, migration, and invasion	Suppresses expression	Directly targets SALL4	Yan et al. (2018)
miR-15a	HCC	Inhibits survival, migration, and invasion	Suppresses expression	Targets SALL4 in vitro	Ma et al. (2021); Zhao et al. (2019); Yin et al. (2019); Xie et al. (2021)
miR-497	HCC	Inhibits self-renewal, metastasis	Suppresses expression	Directly targets SALL4	Zhao et al. (2019)
miR-296-5p	HCC	Inhibits the stemness potency	Suppresses expression	Directly targets BRG1, which binds to the SALL4 promoter	Shi et al. (﻿^2020^)

**Table 2:** Downstream targets of SALL4 in GIT cancers

Targets	Tumor	Function	Action	Signaling Pathways	References
SOX2	ESCC	Promotes invasion and metastasis	Unconfirmed	NOTCH pathway	Forghanifard et al. (2021)
TGF-β1	GC	Induces EMT	Direct	TGF-β/SMAD pathway	Zhang et al. (2018)
HK-2	GC	Promotes glycolysis, accelerates GC progression, leads to poor prognosis	Direct	Cell adhesion, glycolysis, gluconeogenesis, calcium signaling pathway	Shao et al. (2020)
TRIB3	GC	Promotes cancer progression	Unconfirmed	Wnt/β-catenin pathway	Yang et al. (2021a)
CD44	GC	Promotes cell proliferation, migration and invasion	Direct	ERK, STAT3 and NF-κB pathways	Yuan et al. (2016)
VEGF	GC	Promotes angiogenesis	Direct	Unconfirmed	Abouelnazar et al. (2023a)
Gli-1	CRC	Promotes cell growth and tumor origination	Unconfirmed	Unconfirmed	Cheng et al. (2015b)
Bcl-2	CRC	Inhibits tumorigenesis	Unconfirmed	Unconfirmed	Hesari et al. (2019)
β-Catenin	ESCC, CRC, HCC	Promotes proliferation, invasion, metastasis, correlates with liver cirrhosis and an advanced clinical stage	Unconfirmed Direct Unconfirmed	Wnt/β-catenin pathway	He et al. (2016); Hao et al. (2016); Wang et al. (2019b)
miR-146a-5p	HCC	Promotes tumor development by switching the dysfunction of T cells	Direct	Unconfirmed	Yin et al. (2019)
PTEN	HCC	Suppresses tumor progression	Unconfirmed	PI3K/AKT pathway	Tang et al. (2022)
OXPHOS-related genes	HCC	Increase oxygen consumption, mitochondrial membrane potential, and ATP generation	Direct	Unconfirmed	Tan et al. (2019)
EpCAM, KRT19, CD44	HCC	As an EMT and stem cell marker, promotes invasion and spheroid formation	Unconfirmed	EMT signaling pathway	Zeng et al. (2014)
HDAC	HCC	Promotes proliferation	Unconfirmed	Unconfirmed	Zeng et al. (2014)
KDM3A	HCC	May regulate the heterochromatin and cell death (needs to be verified)	Direct	Unconfirmed	Kong et al. (2021)
FoxM1	PDAC	Modulates metastasis efficiency	Unconfirmed	ERK1/2 phosphorylation	Yong et al. (2013); Huynh et al. (﻿^2018^)

## Functions of SALL4 in gastrointestinal tract cancers

SALL4 was first described in humans as an oncogene in 2006 (Ma et al. 2006), after which it was extensively studied and discussed (Zhang et al. 2015; Sun et al. 2022; Dirican and Akkiprik 2016; Moein et al. 2022; Liu et al. 2021; Abouelnazar et al. 2023b; Nicolè et al. 2017; Tatetsu et al. 2016; Yang 2018; Oikawa et al. 2013). It is known that SALL4 has 2 isoforms, SALL4A and SALL4B. SALL4A originates from the full-length transcript, while the spliced isoform SALL4B lacks a part of exon 2 (Milanovich et al. 2015). In murine embryonic stem (ES) cells, SALL4A and SALL4B have different binding sites, with few overlapping sites, playing distinct roles in the maintenance of the pluripotent state (Rao et al. 2023). As SALL4B is the predominant isoform in murine hematopoietic stem cells and progenitors, its overexpression results in a failure of engrafting transplanted bone marrow and reconstituting hematopoiesis (Milanovich et al. 2015). The OCT4 promoter and KRLR sequence have been identified as the transcriptional target and bona fide nuclear localization signal for SALL4B, respectively (Wu et al. 2014). In addition, massive expansion of CD34^+^ cells has been considered one function of SALL4B (Shen et al. 2016). SALL4A interacts with JARID2, PRDM14 and ESRRB, achieving rapid and effective somatic reprogramming (Iseki et al. 2016). In mouse ES cells, SALL4A occupies enhancers and regulates the expression of developmental genes as a 5-formylcytosine binder (Xiong et al. 2016).

Currently, there are extremely limited studies on the exact roles of SALL4A and SALL4B in cancers. In acute lymphoblastic leukemia (ALL), SALL4A may be correlated to the poor prognosis (Peng et al. 2017). SALL4B transgenic mice develop acute myeloid leukemia (AML), which indicates SALL4B contribution to leukemia development and maintenance (Li et al. 2013). SALL4B may be involved in angiogenesis in GC cells by targeting VEGF (Abouelnazar et al. 2023a). Considering the important role of SALL4 in GIT cancers, additional in-depth studies can be performed to explore the functions of the different SALL4 isoforms in cancers.

Since being discovered, SALL4 has been considered to be a biomarker and to play a vital role in the proliferation, apoptosis, invasive migration, epithelial-mesenchymal transition (EMT), chemoresistance, and the maintenance of cancer stem cells (CSCs) through various pathways, such as the NOTCH, Wnt/β-catenin, ERK, STAT3, NF-κB, TGF-β/SMAD, and PTEN/AKT signaling pathways (Zhang et al. 2015; Dirican and Akkiprik 2016; Ma et al. 2006; Nicolè et al. 2017; Tatetsu et al. 2016; Yang 2018; Oikawa et al. 2013). In recent years, an increasing number of studies have focused on the carcinogenicity of SALL4 and its corresponding mechanisms in GIT cancers (Sun et al. 2022; Moein et al. 2022; Liu et al. 2021; Abouelnazar et al. 2023b), and new functions and regulators/targets of SALL4 have been discovered. A recent study has found that SALL4 plays an important role in angiogenesis by transcriptionally regulating VEGF expression (Abouelnazar et al. 2023a). SALL4 is associated with clinicopathological features related to GC progression, and it functions via the Wnt/β-catenin pathway (Yang et al. 2021a), which can be mediated by dual regulation of SALL4 by EZH2 and KDM6A (Ren et al. 2023). Nicotine promotes the stabilization and expression of SALL4 by upregulating the RNA-binding protein interleukin enhancer binding factor 2 (ILF2), which facilitates tumor initiation in esophageal cancer cells (Li et al. 2021). SALL4 induces the invasion and metastasis of colon adenocarcinoma (COAD) and is significantly correlated with TNM grading, histological grading, and lymphatic metastasis in tumor tissues (Zhang et al. 2022). SALL4 activates the PI3K/AKT signaling pathway by targeting PTEN, thereby facilitating the migration, invasion and proliferation of HCC cells (Tang et al. 2022).

There are reviews demonstrating the functions and regulatory mechanisms of SALL4 (Zhang et al. 2015; Sun et al. 2022; Moein et al. 2022; Liu et al. 2021; Abouelnazar et al. 2023b; Nicolè et al. 2017), many of which show that each pathway is involved in many different types of cancers. In the next section, we will focus on GIT cancers, including the most recent findings regarding the role of SALL4 and its mechanisms. We will separately summarize the identified upstream regulators and downstream targets/pathways of SALL4 in each type of GIT cancer, which may help gain an insight into the regulatory mechanisms of SALL4 in each GIT cancer.

## Regulators and targets of SALL4 in gastrointestinal tract cancers

### Esophageal cancer

**Fig. 1:**
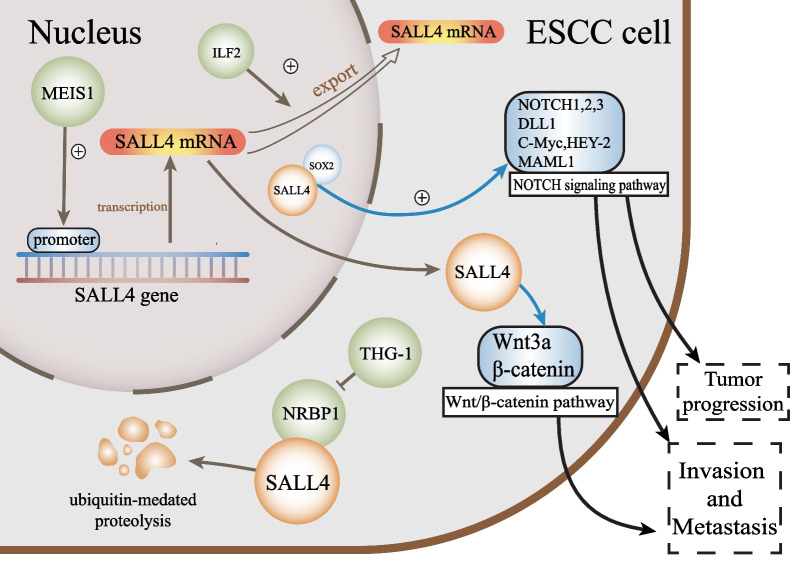
Mechanisms underlying SALL4 regulation and function in esophageal cancer. THG-1 acts as an upstream regulator and inhibits NRBP1-induced ubiquitination of SALL4. MEIS1 downregulation significantly reduces the mRNA expression of SALL4. ILF2 facilitates the nuclear mRNA export of SALL4. SALL4 modulates the Notch and Wnt/β-catenin signaling pathways, promoting the progression, invasion and metastasis in esophageal cancer

In 2020, it was estimated that 604,100 people were diagnosed with esophageal cancer (EC) globally, which resulted in approximately 544,100 deaths (Thrift and El-Serag 2020). Esophageal squamous cell carcinoma (ESCC) is much more common than esophageal adenocarcinoma (EAC). We focused on summarizing research on ESCC, since it accounts for 90% of all cases (Smyth et al. 2017). SALL4 is overexpressed in ESCC tissues (He et al. 2016; Zargari et al. 2020; Yang et al. 2007; Forghanifard et al. 2014), which indicates its involvement in ESCC progression and reveals the underlying function of SALL4 as a predictor in the early diagnosis and therapy of ESCC (Fig. 1).

#### Upstream regulatory genes

Previous research has shown that nuclear receptor-binding protein 1 (NRBP1) downregulates SALL4 protein expression by driving its ubiquitination and proteasomal degradation (Hwang et al. 2020). TSC22 homologous gene-1 (THG-1) interrupts the ubiquitination of SALL4 by competitive binding to NRBP1 (Hwang et al. 2020). Knockdown of THG-1 limits the growth of TE13 cells, a human ESCC cell line, and induces downregulation of SALL4. Conversely, exogenous SALL4 expression significantly restores the growth of the knockdown cell line (Hwang et al. 2020).

By analyzing gene expression patterns and conducting clinicopathological tests on tumor and adjacent tumor-free tissues from 50 ESCC patients, researchers have shown that SALL4 expression positively correlates with that of MEIS1, a homeobox transcription factor (Zargari et al. 2020). Silencing of MEIS1 induces a striking decline in SALL4 expression (Zargari et al. 2020). These results reveal the role of MEIS1 as an upstream regulatory gene of SALL4, and further exploration is needed to determine the downstream pathway of MEIS1/SALL4 in ESCC.

Nicotine-induced upregulation of the RNA-binding protein interleukin enhancer-binding factor 2 (ILF2) promotes the stabilization and expression of SOX2, NANOG, and SALL4 by facilitating nuclear mRNA export and inhibiting hMTR4-mediated degradation, which enhances the stemness and tumor-initiating capacity of esophageal cancer cells (Li et al. 2021).

#### Downstream signaling pathways

Esophageal tumor tissues exhibit elevated expression of both SALL4 and SOX2, which function as stemness markers (Forghanifard et al. 2014). A study has demonstrated the interaction between SOX2 and SALL4 in nuclear protein complexes (Cox et al. 2010). The SOX2/SALL4 stemness axis has been shown to modulate the Notch pathway (Forghanifard et al. 2021). The Notch cascade cell signaling pathway contributes to the pathological process of ESCC, which is modulated by ligands binding to Notch receptors (NOTCH1, 2, and 3), a Notch ligand (DLL1), Notch target genes (C-MYC and HEY2) and a Notch signaling pathway transcription activator (MAML1) (Forghanifard et al. 2015). The expression of these molecules is associated with various pathological properties, such as tumor progression, lymph node metastasis and invasion to the adventitia (Forghanifard et al. 2021).

The Wnt/β-catenin pathway promotes transcriptional changes, driving epithelial-mesenchymal transition (EMT) in cancer and contributing to metastasis (Tang et al. 2020; Yu et al. 2021). Applying the Wnt agonist HLY78 has been shown to improve the motility of ESCC cells (Chen et al. 2022). Previous research has indicated that SALL4 knockdown attenuates EMT and downregulates the expression of Wnt3a and β-catenin in ESCC (He et al. 2016). These findings suggest that SALL4 may be involved in ESCC oncogenesis via the Wnt/β-catenin pathway.

Aldehyde dehydrogenase 1A1 (ALDH1A1) is a CSC marker that is highly expressed in ESCC tissues. ALDH1A1 could activate the AKT signaling pathway via stimulation of AKT phosphorylation, further increasing cancer stem cell-like properties (Wang et al. 2020). Bioinformatics analysis has shown an interaction between the SALL4 and ALDH1A1 genes, and high coexpression of SALL4/ALDH1A1 in serous ovarian carcinoma is significantly associated with distant metastasis and aggressive tumor behavior (Sharbatoghli et al. 2022). These findings may provide a new idea for exploring potential targets of the SALL4/ALDH1A1/AKT signaling pathway.

Caspase-8 overexpression is considered a predictor of worse prognosis of ESCC (Chai et al. 2023). SALL4 regulates multiple caspase family members in tumor cells (Sun et al. 2022; Yong et al. 2013; Chai et al. 2023). The activity of caspase-3/8 in SALL4 knockout cells is elevated in acute B-cell lymphoblastic leukemia (Ueno et al. 2014), indicating that SALL4 maintains the survival of tumor cells by inhibiting caspase family members.

These results reveal roles of the Notch, Wnt/β-catenin, AKT, and caspase-induced apoptosis signaling pathways as potential targets of SALL4 in esophageal cancer. However, their exact functions in this cancer and the underlying mechanisms require further investigation.

### Gastric cancer

**Fig. 2:**
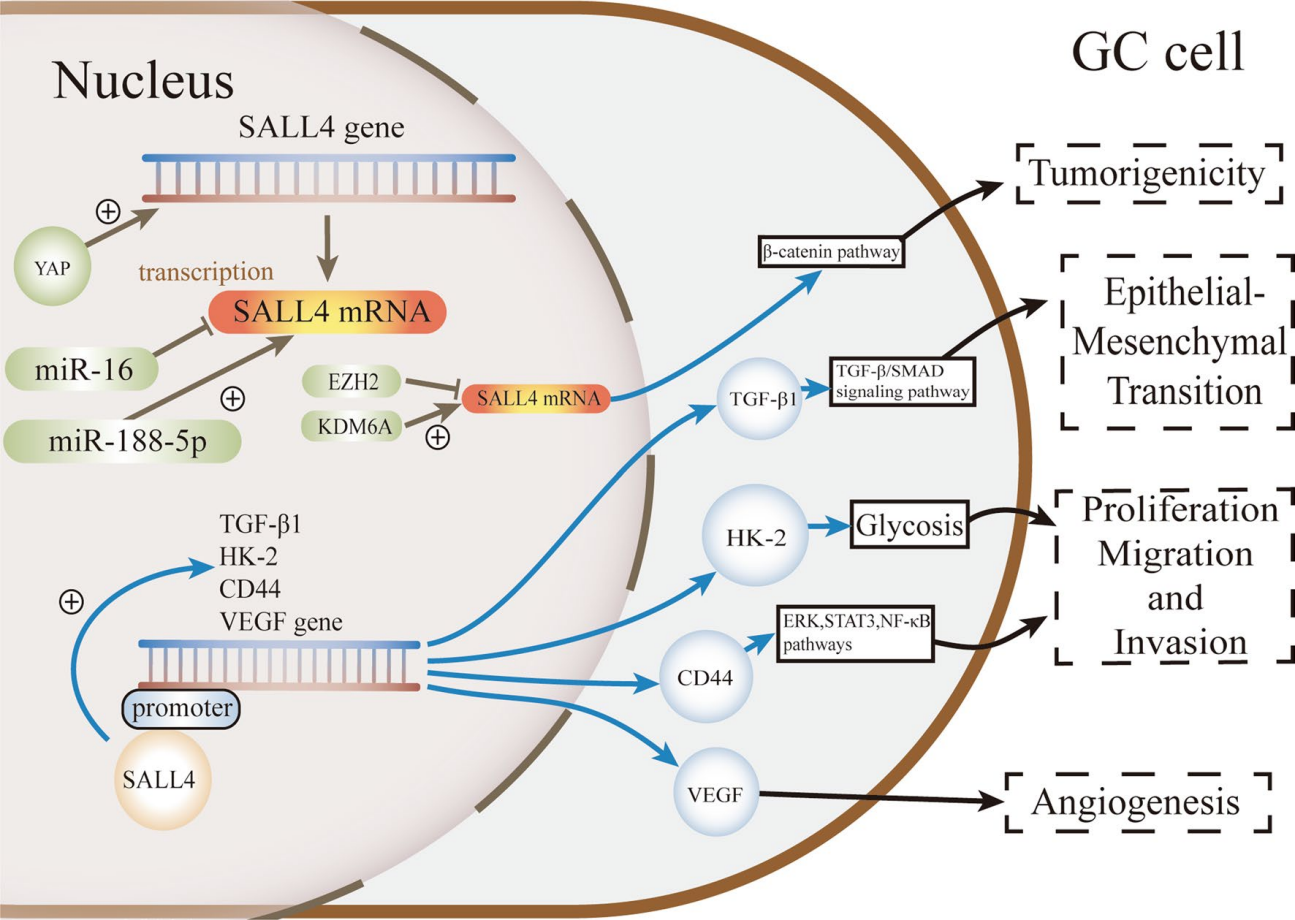
Mechanisms underlying SALL4 regulation and function in gastric cancer. YAP increases the transcription of SALL4. miR-16 downregulates the mRNA level of SALL4, while miR-188-5p expression positively correlates with SALL4 expression. SALL4 targets TGF-β1 and activates the TGF-β/SMAD signaling pathway. HK-2 is another downstream target of SALL4, which promotes glycolysis in gastric cancer. In gastric cancer, CD44 and VEGF are also the downstream targets of SALL4, and are responsible for the oncogenic roles and angiogenesis respectively. Dual-regulated of EZH2 and KDM6A on SALL4 modulates tumor progression via Wnt/β-Catenin pathway in GC

There were over one million new cases of gastric cancer (GC) reported in 2020 (Sung et al. 2021). Advanced-stage GC, lymph node metastasis, noncardia localization, and vascular invasion are associated with a higher SALL4-positive rate (Yang et al. 2021a). Together with alpha-fetoprotein (AFP) and glypican-3 (GPC3), SALL4 is a proven indicator of poor prognosis in GC (Wang et al. 2021a). Both the serum level and immunohistochemical expression of AFP can be used to indicate the poor prognosis for gastric adenocarcinoma, while the SALL4 immunohistochemistry can also be an indicator of adverse prognosis for such cancer (Wang et al. 2021a). Recently, SALL4 helps to identify clinically and molecularly distinct genomic consensus subtypes (CGSs) (Jeong et al. 2023). Besides, positivity of SALL4 can aid in the diagnosis of hepatoid adenocarcinoma of stomach (HAS), a special subtype of gastric cancer with poor prognosis (Yang et al. 2024). Current treatments for GC, including conventional therapy, immunotherapy and targeted therapy, show some curative effects, and clarifying the molecular pathogenesis and regulatory genes underlying GC is necessary for developing novel and personalized treatments for patients. In this section, we will discuss the regulatory genes of SALL4 in GC and its downstream signaling pathways (Fig. 2).

#### Upstream regulatory genes

Yes-associated protein 1 (YAP) expression increases in GC spheroid cells and promotes cell self-renewal (Bie et al. 2020). Immunofluorescence staining has revealed the colocalization of YAP and SALL4 (Bie et al. 2020), and interfering with YAP expression significantly suppresses the mRNA level of SALL4 in MGC-803 cells (Bie et al. 2020). It is necessary to confirm the correlation between YAP and SALL4 in vivo and determine whether the regulatory effect of YAP on SALL4 expression is direct.

The microRNA (miRNA) family modulates biological processes in cancer by targeting different genes (Ishiguro et al. 2014; Ali Syeda et al. 2020; Peng et al. 2021). miR-188-5p is highly expressed in various GC cell lines and promotes the expression of SALL4, thereby facilitating the proliferation and migration of GC cells (Wang et al. 2019a). In contrast, another in vitro study has revealed the anticancer effect of miR-188-5p by targeting ZFP91 (Peng et al. 2018), suggesting that the precise function of miR-188-5p still needs to be confirmed. As a tumor suppressor, miR-16 is expressed at low levels in GC tissues and specifically targets SALL4 at both the RNA and protein levels in GC cells (Jiang and Wang 2018).

According to a study on hepatocellular carcinoma (HCC) (Zhao et al. 2019), miR-497 directly targets SALL4 and negatively regulates its expression, contributing to the stemness properties and metastatic potential of HCC cells (Zhao et al. 2019). Although miR-497 suppresses GC tumorigenesis and progression (Zhang et al. 2021), it is still unknown whether SALL4 participates in the regulation of miR-497 in gastric cancer. Based on these findings, SALL4 is the only potential target gene of miR-497 in gastric cancer, as has been revealed in HCC cells.

#### Downstream signaling pathways

The Wnt/β-catenin signaling pathway, which has been shown to play a role in the regulation of SALL4 in ESCC, may also be involved in this process in GC (Yang et al. 2021a). A study of 1815 GC patients found that SALL4 expression was positively correlated with TRIB3, a core gene in the Wnt/β-catenin pathway (Yang et al. 2021a). The expression of CTNNB1 (also known as catenin beta 1 or β-catenin) is directly upregulated by SALL4 in cervical cancer cells (Chen et al. 2019). Activation of CTNNB1 transcription may give rise to anoikis resistance and enhance the metastatic ability of GC cells (Ye et al. 2020).

Overexpressing the SALL4 gene upregulates the expression of EMT inducer genes, thereby promoting EMT in GC cells (Zhang et al. 2018; Du et al. 2023). Further research has shown that SALL4 modulates EMT by regulating TGF-β1 expression and SMAD phosphorylation (Zhang et al. 2018). These results indicate that SALL4 may activate the TGF-β/SMAD signaling pathway to induce EMT. A recent study has also shown that SALL4 facilitates EMT (Du et al. 2023). SALL4 overexpression promotes the proliferation and invasiveness of GC cells through EMT-related genes, while the knockdown of SALL4 inhibits this process (Du et al. 2023). In addition, the HDAC inhibitor entinostat partially targets SALL4 and suppresses the proliferation, migration, and invasion of gastric cancer cells by regulating the expression of EMT-associated proteins (Du et al. 2023).

SALL4 knockdown downregulates the expression of stemness- and EMT-related genes (OCT4, SOX2, NANOG, C-MYC and CD44) and suppresses the activation of the ERK, STAT3 and NF-κB pathways, thus inhibiting the proliferation and migration of gastric cancer cells (Yuan et al. 2016). As CD44 is a cell adhesion molecule, its overexpression antagonizes SALL4 knockdown-induced inhibition of proliferation, migration, invasion and growth of GC (Yuan et al. 2016). These results suggest that CD44 is partially responsible for the oncogenic roles of SALL4 in GC.

In addition to these signaling pathways, there are other genes and molecules that are considered targets of SALL4 in GC. For example, SALL4 knockdown eliminates the suppressive effect of miR-188-5p on PTEN expression (Wang et al. 2019a), indicating that SALL4 is a key regulatory factor through which miR-188-5p inhibits PTEN.

Abnormal glycolysis contributes to tumorigenesis, metastasis, and drug resistance of cancers, including GC (Ma et al. 2015; Rosa et al. 2015). SALL4 overexpression promotes glycolysis in GC, leading to cell proliferation. SALL4 knockdown or overexpression downregulates or upregulates, respectively, the activity of the HK-2 gene, which is involved in glycolysis (Shao et al. 2020). In addition, knockdown of HK-2 inhibits the promotional effect of SALL4 on glycolysis and its effects on GC cells (Shao et al. 2020), confirming the role of HK-2 as a target gene of SALL4 in GC.

In addition to tumorigenesis, metastasis, and drug resistance of cancers, SALL4 transcriptionally regulates the expression of VEGF to promote angiogenesis (Abouelnazar et al. 2023a). Overexpression of SALL4B increases VEGF-A, −B, and −C gene expression, while SALL4B knockdown reduces their expression (Abouelnazar et al. 2023a).

### Colorectal cancer

**Fig. 3:**
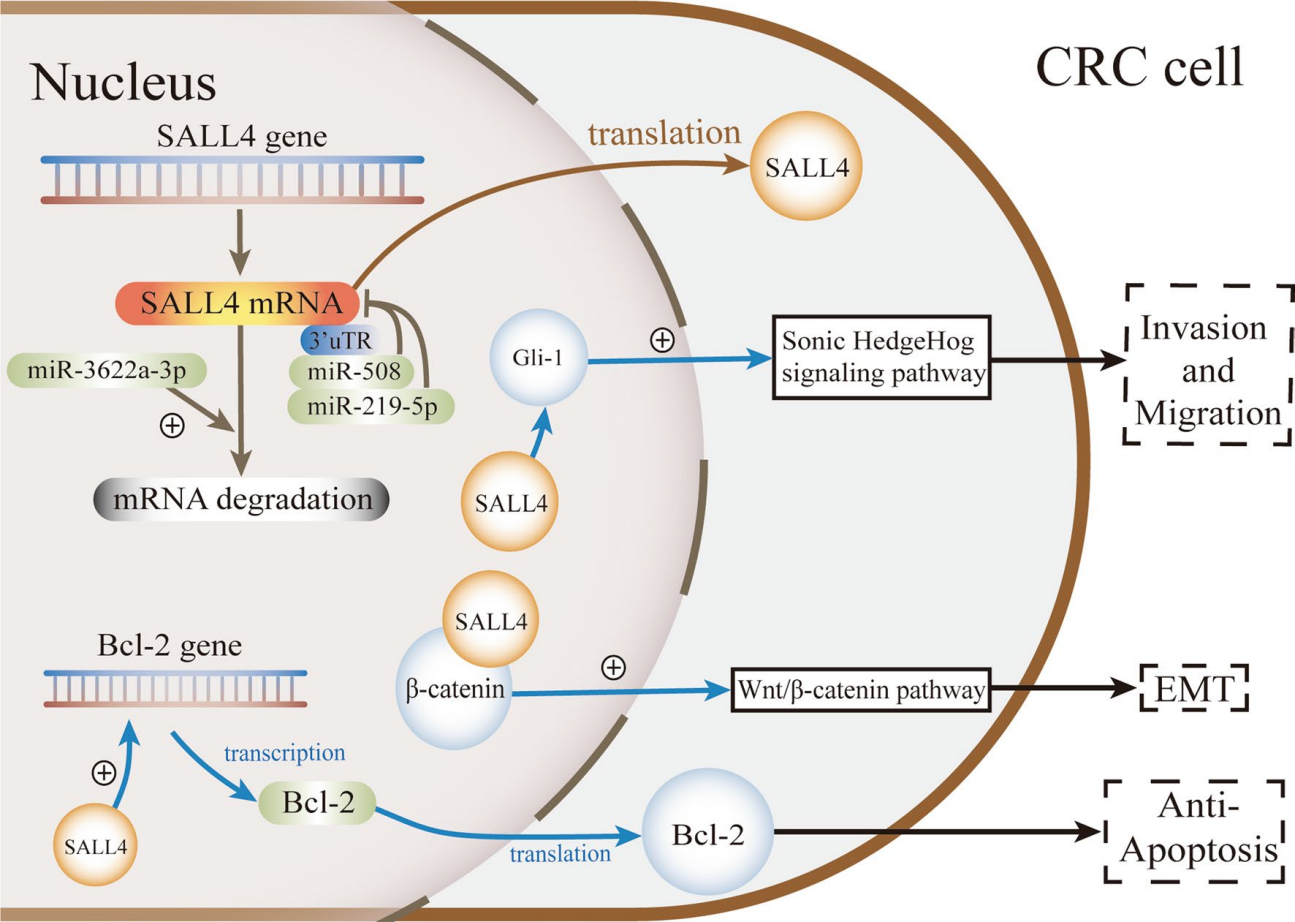
Mechanisms underlying SALL4 regulation and function in colorectal cancer. In colorectal cancer, miR-508 and miR-219-5p suppress SALL4 expression by interacting with its 3′ UTR sequence, while miR-3622a-3p promotes SALL4 mRNA degradation as a tumor suppressor. SALL4 knockdown in CRC cells inhibits Bcl-2 expression and induces cell apoptosis. In CRC cells, SALL4 regulates Gli1 promoting the invasion and migration. SALL4 also targets β-catenin in colorectal cancer, further activating the Wnt/β-catenin pathways

Colorectal cancer (CRC) is ranked second in terms of mortality and third in terms of the incidence as of 2020 (Sung et al. 2021). People with a family history of CRC among first-degree relatives are usually considered high-risk populations (Siegel et al. 2020). This suggests the important role of genetic factors in CRC. It is usually considered that the pathological transformation of CRC results from molecular events involving various pathways (Gryfe et al. 1997). SALL4 expression in CRC tissue and serum correlates with the degree of lymph node metastasis (LNM), differentiation degree, and staging, and patients with high SALL4 expression usually have a shorter mean survival time than those with low SALL4 expression (Hao et al. 2016; Moein et al. 2022; Liu et al. 2021; Abouelnazar et al. 2023b; Wu et al. 2017; Forghanifard et al. 2013). These findings reveal the potential of SALL4 for developing antitumor targets in CRC. The activated pluripotency transcriptional network, consisting of SALL4/OCT4/DPPA2/Nanog in CRC, plays essential roles in the maintenance of the stemness state, self-renewal characteristics, and progression of tumor cells, leading to an increased depth of invasion (Ghodsi et al. 2015). Colorectal adenocarcinoma with enteroblastic differentiation (CAED), a rare malignancy, is also SALL4 positive (Murakami et al. 2019; Abada et al. 2022; Minato et al. 2023). According to a study that examined 46 normal colonic and small intestinal mucosal samples, no SALL4 expression was detected in the normal intestinal epithelium (Inaguma et al. 2017). Therefore, SALL4 has been considered a potential diagnostic and prognostic marker of colorectal cancer (Ardalan Khales et al. 2015; Wu et al. 2017; Yamashiro et al. 2020; Sajadi et al. 2022). In contrast to the above studies, one study showed negative expression of SALL4 in 20 cases of AFP-producing colorectal cancer (Ren et al. 2019), which indicates that the use of SALL4 as a biomarker in this type of CRC needs to be further validated. An immunohistochemistry study on clinical samples revealed that the regulatory transcriptional network of SALL4/OCT4/DPPA2/NANOG had an essential role in the maintenance of the stemness state and self-renewal of CRC cells (Ghodsi et al. 2015). Although the mechanism underlying SALL4 regulation in CRC is not well defined, some clues have been partially uncovered (Fig. 3).

#### Upstream regulatory genes

microRNAs participate in the upstream regulation of SALL4 in CRC (Chang et al. 2020; Cheng et al. 2015a; Yan et al. 2018). In vitro studies have identified miR-3622a-3p as a tumor suppressor by targeting SALL4 (Chang et al. 2020). Researchers have validated that miR-3622a-3p is a microRNA with the lowest expression in 619 CRC specimens and is negatively correlated with worse prognosis using the TCGA database and tumor tissues (Chang et al. 2020). Similarly, miR-219-5p is reduced in malignant tissues with SALL4 overexpression and can interact with the 3' UTR sequence of SALL4 (Cheng et al. 2015a). Research has also shown that miR-219-5p suppresses cell growth, induces apoptosis of CRC, and decreases drug resistance to fluorouracil and oxaliplatin by inhibiting the oncogene SALL4 (Cheng et al. 2015a). Additionally, miR-508 has been shown to decrease the expression of stemness genes in CRC, with SALL4 being one of the genes most downregulated by miR-508 (Yan et al. 2018).

NRBP1 overexpression in CRC cells leads to caspase-dependent intrinsic apoptosis, inhibiting cell proliferation and colony formation (Liao et al. 2018). Whether this inhibitory effect on tumor cells is due to SALL4 targeting, as observed in ESCC (Hwang et al. 2020), requires further exploration.

In addition to the high expression of SALL4, a high frequency of methylation is found in colorectal laterally spreading tumors (LSTs) and protruding adenomas. However, it is unclear whether inactivation of the SALL4 gene by methylation contributes to the development of colorectal adenomas (Sugai et al. 2016). Another study has shown that the SALL4 gene is more frequently hypermethylated in aneuploid cancers (8 of 16, 50%) than in diploid cancers (3 of 18, 17%) (Habano et al. 2007). These results indicate that epigenetic silencing of SALL4 may be associated with tumor cell aneuploidy, which affects the chromosomal stability in intestinal epithelial cells (Habano et al. 2007).

#### Downstream signaling pathways

The Sonic Hedgehog (SHH) signaling pathway regulates tumor origination and cell growth, and Gli-1 is considered the downstream transcription factor of this pathway (Cheng et al. 2015b). The knockdown of SALL4 downregulates the expression of Gli-1 and inhibits oncogenesis in CRC cells, which is antagonized by upregulating Gli-1, suggesting that Gli-1 may be a target gene of SALL4 in CRC (Cheng et al. 2015b). Aberrant activation of the SHH signaling is also responsible for the tumorigenesis in medulloblastoma (MB) (Wang et al. 2018). By forming a trimeric complex with Gli-1 and HDAC1, SALL4 potentiates Gli-1 transcriptional activity therefor sustains SHH-MB cells proliferation (Lospinoso Severini et al. 2024). These findings highlight SALL4 as a crucial role in SHH pathway and promising therapeutic target in SHH-dependent cancers.

SALL4 mediates EMT in CRC cells (Zhang et al. 2022), as in other gastrointestinal tract cancers (He et al. 2016), with the Wnt/β-catenin pathway potentially involved in this process (Chang et al. 2020). SALL4 expression correlates with the levels of TRIB3 (Yang et al. 2021a), which directly binds to β-catenin in CRC (Hua et al. 2019). The enhancement of canonical Wnt signaling in CRC cells increases SNAIL expression (a zinc finger transcription factor family that drives EMT), which regulates EMT by inhibiting E-cadherin and promotes local invasion (Goossens et al. 2017). Immunofluorescence and coimmunoprecipitation show the colocalization of SALL4 and β-catenin and indicate their interaction in human CRC tissues and cell lines (Hao et al. 2016). In addition, the function of SALL4 in promoting lymph node metastasis and an advanced TNM stage may be partly related to its interaction with β-catenin and subsequent aberrant activation of the Wnt/β-catenin signaling pathway (Hao et al. 2016). These studies suggest that the Wnt/β-catenin pathway is a downstream target of SALL4 in CRC.

In the regulation of cancer cell apoptosis, the interaction among Bcl-2 family members, for example, Bcl-2 and Bax, plays a significant role (Chipuk et al. 2010). Bcl-2 overexpression suppresses cell apoptosis and downregulates Bax expression, ultimately promoting cell proliferation and impeding programmed cell death. Knockdown of SALL4 effectively inhibits Bcl-2 expression, leading to the induction of cell apoptosis in CRC cells (Hesari et al. 2019). Therefore, SALL4 could promote oncogenesis and inhibit apoptosis in CRC cells by targeting Bcl-2.

### Liver cancer

**Fig. 4:**
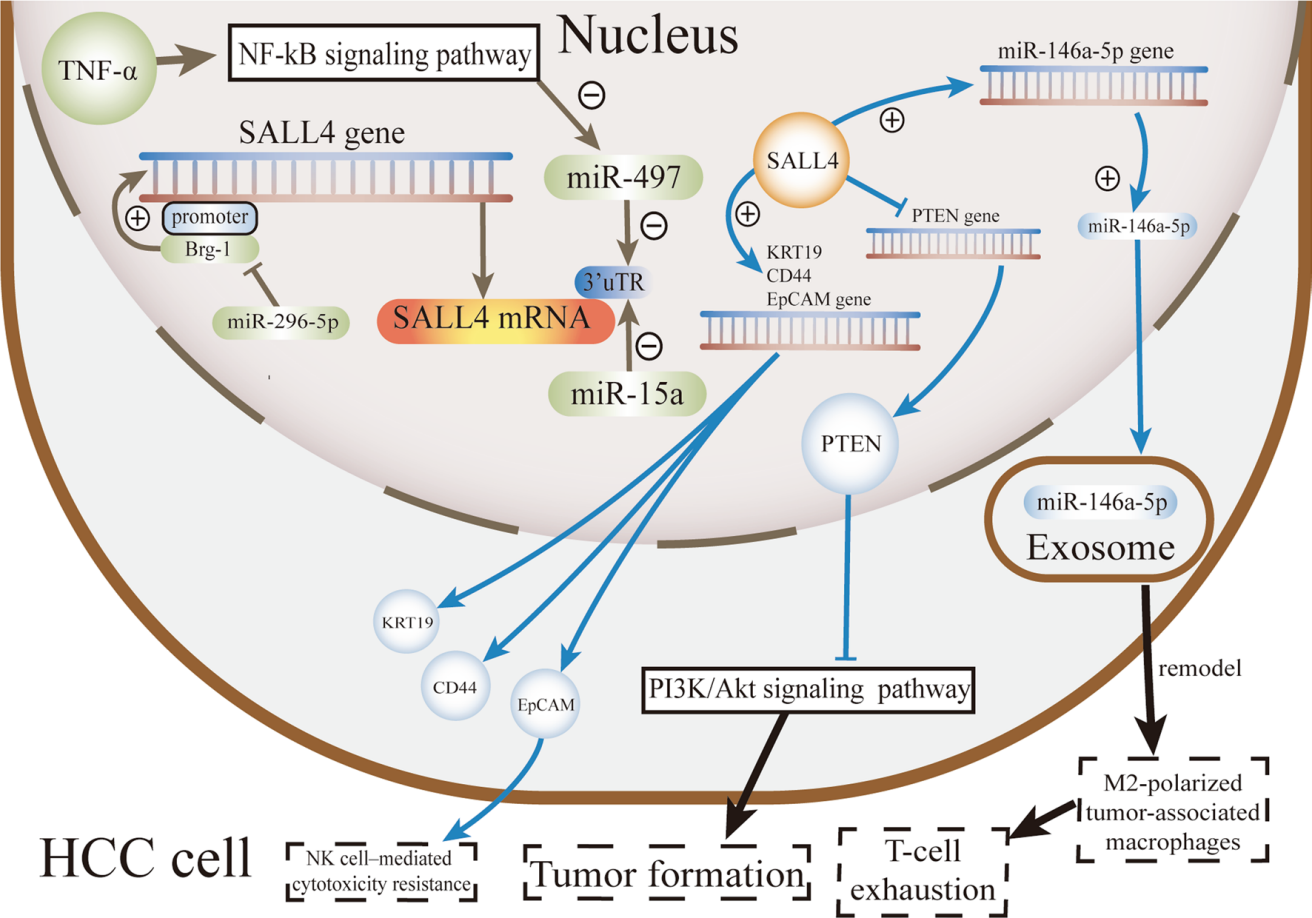
Mechanisms underlying SALL4 regulation and function in liver cancer. SALL4 expression is inhibited by miR-15a, which leads to decreased proliferation, migration, and invasion of HCC cells. TNF-α inhibits miR-497 expression and upregulates SALL4. SALL4 promotes hepatocarcinogenesis by activating the PTEN/AKT pathway and targeting miR-146a-5p, which further promotes cancer development. As a transcription factor, SALL4 up-regulates the hepatic stem cell markers KRT19, EPCAM, and CD44

Owing to its limited therapeutic interventions, liver cancer is the second leading cause of cancer mortality. Hepatocellular carcinoma (HCC) is the most common type of liver cancer. Since most HCC patients can only receive palliative treatments (Galle et al. 2018), studying new targets of chemotherapy drugs is of great significance. As a stem cell-associated gene, SALL4 is highly expressed in fetal liver progenitor cells but not in adult hepatocytes (Yong et al. 2013) and is considered a stem cell biomarker in liver cancers (Oikawa et al. 2013). By examining 124 samples of HCC tissues, 44 samples of adjacent noncancerous cirrhotic tissues and 10 samples of liver hemangioma tissues, researchers found high expression of SALL4 and its relevance to the adverse prognosis of patients with HCC (Yin et al. 2016). PD-L1-positive HCC frequently shows positive expression of SALL4 (Nishida et al. 2020). Additional studies have also shown that SALL4 mRNA and protein levels are elevated in HCC tissues compared with adjacent tissues (Wang et al. 2017a, 2021b; Moeini et al. 2017; Tanaka et al. 2015). Clinicopathological analysis has revealed that patients with high SALL4 expression in HCC have a worse prognosis than those with low expression (Zeng et al. 2014; Wang et al. 2017; Leake 2013; Jung et al. 2022). SALL4-positive HCC patients exhibit higher HBs antigen positivity and higher levels of tumor markers (Wang et al. 2017). Activated SALL4 has been found in HCC with high metabolic activity, which displays the features of poor survival, the strongest stem cell signature, high genomic instability, and low potential for benefiting from immunotherapy (Jung et al. 2022). SALL4 regulates the stemness of EpCAM-positive HCC as a transcription factor and is associated with high values of serum alpha-fetoprotein, a high frequency of hepatitis B virus infection, and poor prognosis after surgery (Zeng et al. 2014). Another study has shown that EpCAM^high^ liver cancer stem cells can resist NK cell–mediated cytotoxicity (Park et al. 2020). In hepatoblastoma, high expression of SALL4 is also associated with poor prognosis (Zhou et al. 2016). To treat HCC, small-molecule drugs targeting SALL4 have been developed. Competitive inhibitors of SALL4 have been shown to block its carcinogenic effects both in vitro and in vivo (Jones 2013). Information on the underlying mechanisms of SALL4 regulation in HCC has been reported (Fig. 4).

#### Upstream regulatory genes

miR-15a inhibits the expression of SALL4, which in turn accelerates apoptosis and suppresses the oncogenic potential of HCC cells, such as proliferation, migration, and invasion (Ma et al. 2021). miR-497 also inhibits SALL4 expression and suppresses the self-renewal and metastasis of HCC cells by directly targeting SALL4 (Zhao et al. 2019). Upstream TNF-α downregulates miR-497 expression, upregulates SALL4 expression, and promotes the metastatic phenotype of HCC cells (Zhao et al. 2019). miR-296-5p inhibits the stemness potency of HCC cells through the BRG1/SALL4 axis. miR-296-5p directly targets brahma-related gene-1 (BRG1), which binds to the SALL4 promoter and enhances SALL4 transcription, thereby inhibiting the stemness potency of HCC cells (Shi et al. 2020).

As only a few genes regulating SALL4 have been studied in liver cancer, further research is needed to explore more upstream regulatory genes of SALL4 in this cancer.

#### Downstream signaling pathways

As a tumor suppressor, PTEN (phosphatase and tensin homolog) is considered a target gene of SALL4 (Tang et al. 2022). SALL4 activates PI3K/AKT signaling pathway by mediating PTEN silencing, promoting the development of HCC and leading to a poor prognosis (Tang et al. 2022).

In addition to the PI3K signaling pathway, SALL4/Wnt/β-catenin signaling has been shown to be associated with the clinicopathological features and prognosis of HCC patients (Wang et al. 2019b). Significantly upregulated mRNA and protein expression of SALL4, Wnt3a and β-catenin in HCC tissues is associated with tumor differentiation, the TNM stage, tumor size, vascular invasion and liver cirrhosis in HCC patients (Wang et al. 2019b). The aberrant of Wnt/β-catenin signaling promotes the progression of liver cancer (He and Tang 2020; Wang et al. 2019c), which also determines the responses of hepatoma cells to lenvatinib treatment (Wang et al. 2023).

In HCC-derived exosomes, SALL4 regulates M2 polarization and miR-146a-5p expression, which promotes cancer progression (Yin et al. 2019). miR-146a-5p can be delivered into macrophages and promote them toward M2-polarized tumor-associated macrophages, which impair T-cell functions (Yin et al. 2019). In a DEN/CCL4-induced HCC mouse model, SALL4 directly modulates the expression of miR-146a-5p by binding to its promoter, and blocking the SALL4/miR-146a-5p interaction in HCC downregulates the expression of inhibitory receptors on T cells, reverses T-cell exhaustion, and delays HCC progression (Yin et al. 2019). These findings verify the role of the SALL4/miR-146a-5p axis in HCC.

The conversion of bioenergy metabolism often occurs in the process of tumorigenesis, with a common form of switching from mitochondrial oxidative phosphorylation (OXPHOS) to aerobic glycolysis (Liu and Shyh-Chang 2019). While in HCC cell line, SALL4 binds and upregulates the expression of OXPHOS genes and other mitochondrial genes to increase mitochondrial oxidative phosphorylation, which reveals the metabolic reprogramming function of SALL4 in tumorigenesis (Tan et al. 2019; Liu and Shyh-Chang 2019). It may be possible to find downstream targets of SALL4 by focusing on OXPHOS-related genes in GIT cancers.

An earlier study showed that SALL4 might promote cell proliferation by directly regulating the expression of cyclins D1 and D2 (Oikawa et al. 2013). Although SALL4 increases the expression of EMT genes such as TWIST1, the cell migration and invasion of liver cancer cells are not directly affected (Oikawa et al. 2013). Notably, other researchers have obtained somewhat different results. Activated SALL4 enhances spheroid formation, invasion capacities, and key characteristics of cancer stem cells while upregulating the EMT markers, hepatic stem cell markers KRT19, EpCAM, and CD44, in hepatocyte cell lines. In contrast, knockdown of SALL4 attenuates the invasion and spheroid formation capacities with decreased expression of EpCAM and CD44 in HCC cells (Zeng et al. 2014).

Recently, researchers have identified and validated a DNA-binding domain of SALL4 (an AT-rich motif) through an unbiased screen of a protein-binding microarray (PBM) and cleavage under targets and release using nuclease (CUT&RUN) experiments (Kong et al. 2021). In aggressive liver cancer cells, new target genes that are directly regulated by SALL4 (240 repressed and 190 activated by SALL4) have also been discovered by RNA sequencing analyses. The SALL4-repressed genes include a chromatin modifier gene (KDM3A) and a family of transcription factors (forkhead, BCL, KLF, and TBX5). KDMs are genes encoding a family of histone 3 lysine 9-specific demethylases that regulate the methylation statuses of H3K9 and the chromatin (Kong et al. 2021).

SALL4 induces epigenetic modification in HCC cells. SALL4 positively regulates histone deacetylase (HDAC) activity, and HDAC inhibitors suppress the proliferation of SALL4-positive HCC cells (Zeng et al. 2014).

Pancreatic cancer was responsible for approximately 496,000 new cases worldwide in 2020, ranking 14th among all cancers, with a poor prognosis and high mortality rate, accounting for nearly as many deaths (466,000) (Sung et al. 2021). Over 90% of cases of pancreatic cancer are pancreatic ductal adenocarcinomas (PDACs) (Wood and Hruban 2012), which have a low survival rate due to late diagnosis, highlighting the significance of early diagnosis and the use of biomarkers in the management of pancreatic cancer. The function and regulation of SALL4 in pancreatic cancer have received less attention. A recent study has identified the SALL4^high^ PDAC subset that is associated with the poor prognosis, indicating SALL4 as a potential biomarker in pancreatic cancer (Vienot et al. 2023). New advances have suggested the role for SALL4 in promoting the proliferation and migration of PDAC cells and in regulating mitochondrial ROS levels through the FoxM1/PrxIII axis, which is activated by the phosphorylation of ERK1/2 (Huynh et al. 2018). In pancreatic cancer, TSPAN1 (tetraspanin 1) is upregulated by the activation of Wnt/β-catenin to promote cancer proliferation (Zhou et al. 2021). PVT1-induced gemcitabine resistance is also associated with the activation of Wnt/β-catenin signaling pathway in pancreatic cancer (Zhou et al. 2020). However, whether SALL4 acts via this pathway in pancreatic cancer is still unclear and worth investigating.

## Future perspectives

Although some SALL4-related genes and pathways have been identified in GIT cancers, there are still unknown targets that need to be explored. Notably, there are genes and pathways that are aberrantly expressed or activated in GIT cancers have been identified as upstream regulatory genes or downstream signaling pathways of SALL4 in some cancers other than GIT (Table 3). It is necessary to explore the relationship between SALL4 and these potential targets in GIT cancers. Finding these genes and pathways and understanding the underlying mechanisms may aid in the diagnosis and treatment of gastrointestinal cancers.

**Table 3:** Potential genes/pathways related to SALL4 regulation in GIT cancers

Gene/Pathway	Aberrant expression in GIT cancer	Function in tumor	Relationship with SALL4	References
HOXA11-AS	GC, CRC	Promotes progression	Upregulates SALL4 by sponging miR-3619-5p in NSCLC	Xie et al. (2021); Xia et al. (2021); You et al. (2021); Chen et al. (2020)
miR-3619-5p	GC, CRC	Suppresses progression	Downregulates SALL4 in NSCLC	Xia et al. (2021); Liu et al. (2020); Song et al. (2022)
DNMTs	ESCC, GC, CRC, HCC	Promotes progression	Interacts with SALL4 in HEK293 cell	Fattahi et al. (2018); Yang et al. (2012); Su et al. (2020); Purkait et al. (2022); Luo et al. (2019); Hassouna et al. (2020)
TP53	ESCC, GC, HCC	Suppresses progression	SALL4 interacts with p53 and exerts anti-apoptotic function in mouse ESCs	Zhong et al. (2023); Battista et al. (2021); Sahgal et al. (2021); Khemlina et al. (2017); Guichard et al. (2012); Liebl and Hofmann (2021); Wang et al. (2022b)
MAPK pathway	ESCC, GC, CRC, HCC	Promotes progression	Downstream target of SALL4 in prostate cancer	Fang and Richardson (2005); Shen et al. (2023); Zhang et al. (2020); Yang and Huang (2015); Zheng et al. (2011); Chan et al. (2021); Wang et al. (2022a); Zhou et al. (2023﻿)

A previous study has shown that the oncogenes HOXA11-AS and SALL4 are both upregulated in non-small cell lung cancer (NSCLC) tissues and cells, and HOXA11-AS regulates SALL4 expression by sponging miR-3619-5p (Xia et al. 2021). HOXA11-AS has been shown to be highly expressed and to act as an oncogene via different microRNA pathways in GC (Xie et al. 2021; You et al. 2021) and CRC (Chen et al. 2020). Therefore, exploring the relationship between HOXA11-AS and SALL4, as well as the role of the miR-3619-5p/SALL4 axis in GIT cancers, may provide new targets for therapy.

In glioma, SALL4 promotes cell proliferation by inhibiting PTEN expression, thereby activating the PI3K/AKT signaling pathway (Liu et al. 2017). PTEN, as a tumor suppressor gene, inhibits the PI3K/ AKT pathway in EC, GC, and CRC (Yang et al. 2021b; Liang et al. 2021; Hu et al. 2022). SALL4 has been reported to be an upstream regulatory gene of PTEN in gastric cancer (Wang et al. 2019a) and hepatocellular carcinoma (Yong et al. 2013), but it is unclear whether it has a similar effect on PTEN in esophageal, colorectal, and pancreatic cancer, which needs further investigation.

MAPK pathway plays a role in progression of many diseases including GIT cancers (Fang and Richardson 2005; Shen et al. 2023; Zhang et al. 2020; Yang and Huang 2015; Zheng et al. 2011; Chan et al. 2021; Wang et al. 2022a). A recent study has shown that MAPK pathway is involved in SALL4‐mediated prostate cancer progression (Zhou et al. 2023). These findings indicate that the potential relationship between SALL4 and MAPK pathway in GIT cancers is worth exploring.

TP53 acts a tumor suppressor gene that is frequently mutated in GTI cancers (Olivier et al. 2010; Zhong et al. 2023; Battista et al. 2021; Sahgal et al. 2021; Khemlina et al. 2017; Guichard et al. 2012; Liebl and Hofmann 2021). In mouse embryonic stem cells (mESCs), SALL4 has been shown to interact with p53 and play anti-apoptotic function in a p53-dependent manner (Wang et al. 2022b). It is necessary to investigate if SALL4 exerts its role as a p53-interacting partner.

Epigenetic changes participate in the pathogenesis and development of GIT cancers (Tamura et al. 2000; Fattahi et al. 2018; Lee et al. 2008; Yang et al. 2012). The CDH1 gene, also known as E-cadherin, is usually methylated in gastric cancer (Tamura et al. 2000; Fattahi et al. 2018), which is related to the recurrence of ESCC (Lee et al. 2008). The SALL4 protein can interact with various DNA methyltransferases (DNMTs) and modulate enzyme activities (Yang et al. 2012). Therefore, methylation genes may also be potential downstream targets of SALL4 in GIT cancers, which can be studied in more detail.

In addition, studies of the SALL4 isoforms usually focus on stem cells (Moein et al. 2022; Rao et al. 2023; Wu et al. 2014; Shen et al. 2016; Iseki et al. 2016; Xiong et al. 2016; Cohen et al. 2015). Differences in the functions and regulatory pathways of the SALL4 isoforms in tumorigenesis need further research.

## Conclusion

As an oncogene, SALL4 has been found to be abnormally elevated in both tumor tissues and cells (Ardalan Khales et al. 2015; Cheng et al. 2015b). While the regulation of SALL4 and its targets has not been clearly demonstrated, our review summarizes the currently known functions, upstream regulatory mechanisms, and downstream targets of SALL4 in GIT cancers. This review shows that SALL4 participates not only in the growth, anti-apoptosis, metastasis, and invasion of these cancers but also in drug resistance (Cheng et al. 2015a), EMT (Zhang et al. 2018), OXPHOS (Tan et al. 2019), DNA methylation (Yang et al. 2012) and angiogenesis (Abouelnazar et al. 2023a), in some cases. The expression of SALL4 is regulated by upstream regulators such as THG-1 (Hwang et al. 2020), MEIS1 (Zargari et al. 2020), YAP (Bie et al. 2020), ILF2 (Li et al. 2021), KDM6A and EZH2 (Ren et al. 2023) and some members of the microRNA family (Jiang and Wang 2018; Chang et al. 2020; Cheng et al. 2015a; Yan et al. 2018; Peng et al. 2018). On the other hand, SALL4 acts as an oncogene via the Wnt/β-catenin pathway (He et al. 2016), TGF-β/SMAD pathway (Zhang et al. 2018), Notch pathway (Forghanifard et al. 2021), PI3K/AKT pathway (Tang et al. 2022), ERK, STAT3 and NF-κB pathway (Yuan et al. 2016) in various GIT cancers. Since many potential molecular mechanisms are still unknown, extensively exploring new regulators of SALL4 and its targets as promising biomarkers for the diagnosis and therapy of GIT cancers remains valuable and meaningful.

## Data Availability

Not applicable.
